# Cleavage of the SARS Coronavirus Spike Glycoprotein by Airway Proteases Enhances Virus Entry into Human Bronchial Epithelial Cells *In Vitro*

**DOI:** 10.1371/journal.pone.0007870

**Published:** 2009-11-17

**Authors:** Yiu-Wing Kam, Yuushi Okumura, Hiroshi Kido, Lisa F. P. Ng, Roberto Bruzzone, Ralf Altmeyer

**Affiliations:** 1 HKU-Pasteur Research Center, The University of Hong Kong, Hong Kong SAR, China; 2 Singapore Immunology Network, Agency for Science, Technology and Research (A*STAR), Singapore, Singapore; 3 Department of Nutritional Physiology, Institute of Health Biosciences, University of Tokushima Graduate School, Tokushima, Japan; 4 Division of Enzyme Chemistry, Institute for Enzyme Research, University of Tokushima Graduate School, Tokushima, Japan; 5 Department of Biochemistry, Yong Loo Lin School of Medicine, National University of Singapore, Singapore, Singapore; Université Pierre et Marie Curie, France

## Abstract

**Background:**

Entry of enveloped viruses into host cells requires the activation of viral envelope glycoproteins through cleavage by either intracellular or extracellular proteases. In order to gain insight into the molecular basis of protease cleavage and its impact on the efficiency of viral entry, we investigated the susceptibility of a recombinant native full-length S-protein trimer (triSpike) of the severe acute respiratory syndrome coronavirus (SARS-CoV) to cleavage by various airway proteases.

**Methodology/Principal Findings:**

Purified triSpike proteins were readily cleaved in vitro by three different airway proteases: trypsin, plasmin and TMPRSS11a. High Performance Liquid Chromatography (HPLC) and amino acid sequencing analyses identified two arginine residues (R667 and R797) as potential protease cleavage site(s). The effect of protease-dependent enhancement of SARS-CoV infection was demonstrated with ACE2 expressing human bronchial epithelial cells 16HBE. Airway proteases regulate the infectivity of SARS-CoV in a fashion dependent on previous receptor binding. The role of arginine residues was further shown with mutant constructs (R667A, R797A or R797AR667A). Mutation of R667 or R797 did not affect the expression of S-protein but resulted in a differential efficacy of pseudotyping into SARS-CoVpp. The R667A SARS-CoVpp mutant exhibited a lack of virus entry enhancement following protease treatment.

**Conclusions/Significance:**

These results suggest that SARS S-protein is susceptible to airway protease cleavage and, furthermore, that protease mediated enhancement of virus entry depends on specific conformation of SARS S-protein upon ACE2 binding. These data have direct implications for the cell entry mechanism of SARS-CoV along the respiratory system and, furthermore expand the possibility of identifying potential therapeutic agents against SARS-CoV.

## Introduction

Proteolytic cleavage of the viral envelope glycoprotein into a receptor binding and a fusogenic transmembrane subunit is important to regulate virus entry and infectivity [1]. Previous studies showed that viral glycoprotein activation is mediated by secreted proteases recognizing either monobasic or multibasic cleavage sites [2]. Cleavage of viral glycoprotein has been demonstrated in retrovirus, ortho and paramyxoviruses to regulate virus entry and fusion [3], [4], [5]. The extracellular processing of the envelope glycoprotein has a major impact on the infectivity of virulent or avirulent strains of influenza viruses, Sendai virus and Newcastle disease virus [6], [7], [8]. A typical example is influenza A virus, where virus-cell fusion activity is induced by post-translational proteolytic cleavage of the envelope glycoprotein that is mediated by trypsin-like protease in the bronchial epithelium and airway secretion [9]. Several proteases such as tryptase clara, mini-plasmin, ectopic anionic trypsin, mast-cell tryptase and tryptase TC30, which have been isolated from airway epithelial, can selectivity cleave the consensus cleavage motif of human influenza A virus envelope glycoprotein [10], [11], [12], [13] and determine the virus tropism and infectivity. Recent advance of human genome studies identified a large number of transmembrane serine protease (TMPRSS). Various TMPRSS members with known airway localization have been identified from the respiratory tract. TMPRSS11a, one of the newly identified members of type II transmembrane serine proteases, is expressed in upper respiratory tract (pharynx and trachea) (unpublished data). However, less is known about TMPRSS that activate pneumotropic virus under natural infection.

Although enhancement of virus infection has been demonstrated for bovine (BCoV) and rat (RCV) coronaviruses by treatment of cells with trypsin [14], [15], the precise role of trypsin during coronavirus infection is still unknown. Several members of coronavirus possess a protease cleavage site, which is essential for infectivity and virus-cell membrane fusion, and cleavage at these sites yields two non-covalently linked subunits S1 and S2 [16], [17]. The N-terminal S1 subunit is responsible for receptor binding whereas the membrane-anchored S2 subunit is important for fusion between viral and cellular membranes. Evidence from mouse hepatitis virus (MHV) and BCoV suggested the importance of S protein cleavage into two non-covalently linked S1 and S2 subunits that remain on the virus envelope surface during virus maturation [18], [19]. Uncleaved S protein is functional but cleavage may enhance cell fusion activity and/or virus infectivity [20], [21]. Susceptibility of S protein to cleavage depends on virus strains and host cell types. Similar to group I coronaviruses, sequence analysis suggests that S protein from SARS-CoV is not expected to be cleaved since typical amino acid cleavage sites found in coronavirus group II and III (RRFRR, RRSRR, RSRR, RARS and RARR) are not located in the SARS S protein [22].

Recent findings have suggested the importance of trypsin treatment in activating SARS spike glycoprotein mediated cell-cell fusion [23]. Syncytia formation was observed between SARS spike glycoprotein expressing 293T cells and VeroE6 cells after brief trypsin treatment [24]; trypsin has been shown to induce cleavage of monobasic cleavage site and activate influenza viruses in cell culture system [4], [25]. It is not known whether the functionality of spike glycoproteins is dependent on the activity of trypsin inducing their proteolytic cleavage. Nothing is known about the role of proteases that cleave/modify SARS spike glycoprotein under natural infection.

Conformational reorganization of SARS spike glycoprotein has been demonstrated from cryo-electron microscopic analysis whereby structural transition of the spike glycoprotein has been observed when irradiated SARS-CoV virion binds to the virus receptor, angiotensin-converting enzyme (ACE2) [26]. These experiments showed that receptor-binding and subsequent membrane fusion occur with different phases of structural re-arrangements. Possibly, the protease-modified SARS spike glycoprotein is *de facto* the glycoprotein responsible for virus entry. To address this possibility we have investigated whether cleavage has any significant effect on SARS-CoV entry into airway epithelial cells by using S-pseudotyped lentiviral vectors (SARS-CoVpp) encoding a luciferase reporter gene to mimic SARS-CoV entry. We observed that SARS-CoV spike glycoprotein can be efficiently cleaved by several airway proteases and that this processing enhances entry of SARS-CoVpp. Furthermore, we have identified the putative cleavage sites of airway proteases and, by site-directed mutagenesis, have determined the role of specific amino acid residue for proteolytic processing of the envelope glycoprotein, and for SARS-CoVpp entry into human airway epithelial cells (16HBE) *in vitro*. While this manuscript was still in progress, one of the two natural cleavage sites described here, at position 797, was reported in a separate independent study using only trypsin for cleavage [27]. This study further supports and strengthens the demonstration of the critical role of receptor-dependent cleavage of spike protein by airway proteases, providing deeper insights into the exact mechanism of virus entry enhancement.

## Results

### Susceptibility of Various Human Airway Epithelial Cells to SARS-CoV S Mediated Infection

**Figure 1:**
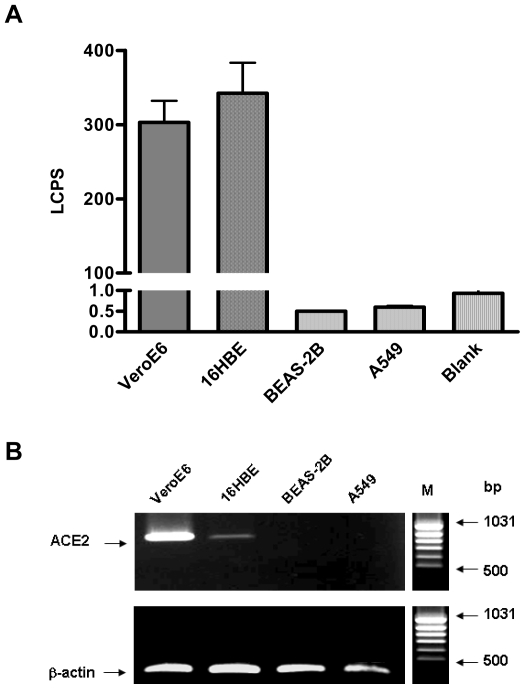
Susceptibility of various human airway epithelial cells to SARS-CoV S-mediated infection. A, VeroE6 (10,000 cells/well), 16HBE, BEAS-2B and A549 (20,000 cells/well) cells were seeded onto 96-well plates 24 h before SARS-CoVpp infection. Pseudotypes (SARS-CoVpp) were collected from culture medium and concentrated as described previously [33]. SARS-CoVpp were incubated with various cell lines and transduction was measured by determination of the luciferase activity expressed as luminescence counts per second (LCPS). VeroE6 cells were used as positive control. All experiments were performed in triplicates and data are presented as means±SE of two or three independent experiments. B, ACE2 expression from various mammalian airway cell lines. Cell lysates were collected and ACE2 RNA molecules were detected by RT-PCR. Amplified ACE2 cDNA products from 16HBE, BEAS-2B and A549 are shown in lanes 2 to 4, respectively. VeroE6 cell line (lane 1) was used as positive control for ACE2 expression. The quantity of total RNA templates was normalized to β-actin expression as shown from the lower panel. Lane M represents the DNA size marker and the size of DNA bands are indicated on the right.

The respiratory tract has been shown to be the primary site for SARS-CoV entry [28], [29]. In order to study the effects of airway protease on SARS-CoV entry mechanism, the susceptibility of various human airway epithelial cell lines were tested for SARS-CoV S-mediated infection. To monitor SARS-CoV entry, we pseudotyped a lentiviral vector with the SARS-CoV Spike glycoprotein (referred to herein as SARS-CoVpp). These recombinant viruses encoding the luciferase reporter gene and expressing the SARS-CoV Spike glycoprotein at the virion surface have been shown to faithfully mimic the SARS-CoV entry process [30]. Three different airway cell lines: 16HBE, BEAS-2B and A549 were analyzed for SARS-CoVpp infection. We observed that only 16HBE cells (human bronchial epithelial cells) were efficiently infected by SARS-CoVpp, with levels of luciferase activity similar to those measured in the positive control cell line VeroE6 (Figure 1A). By contrast, another human bronchial epithelial cell line, BEAS-2B, and a human alveolar cell line, A549 were non-permissive to SARS-CoVpp infection (Figure 1A). The susceptibility to SARS-S mediated infection correlated well with ACE2 expression [31], which was indeed detected by RT-PCR only in 16HBE and VeroE6 but not in A549 and BEAS-2B cells (Figure 1B). These results support previous data showing ACE2 is the functional cellular receptor for SARS-CoV and 16HBE cells were therefore chosen for the subsequent protease cleavage study.

### Proteolytic Modification of Immunopurified Trimeric-S Glycoprotein (TriSpike)

**Figure 2:**
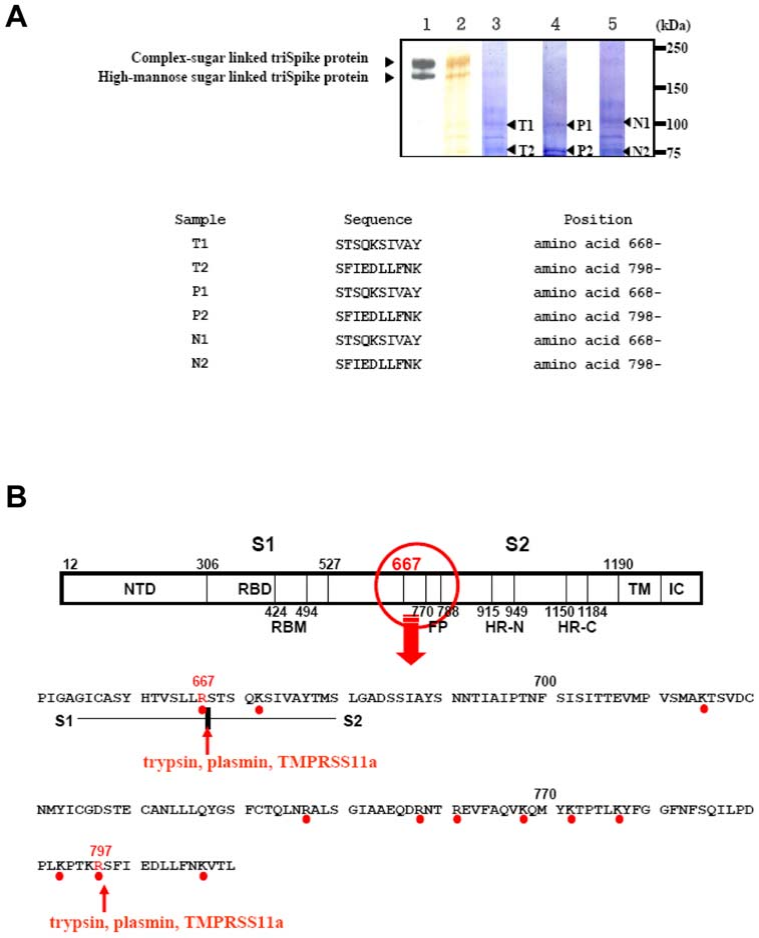
Identification of airway protease cleavage site(s) along the amino acid sequence of SARS-CoV S glycoprotein. A, Purified triSpike proteins (lane 1: detected by Western immunoblot, lane 2: silver staining) were incubated with 0.2 mU of trypsin (lane 3), plasmin (lane 4) or TMPRSS11a (lane 5) identified and purified from lungs and bronchi. Cleavage products were visualized and prepared as described in Materials and Methods. Amino acid sequences (T1, T2, P1, P2, N1, and N2) corresponding to the cleaved triSpike proteins are shown in the lower panel. B, Three different types of airway proteases (trypsin, plasmin and TMPRSS11a) utilize the same amino acid residues for protein cleavage. A schematic diagram representing the amino acid sequence of SARS-CoV S glycoprotein shows on top of the figure. A red circle indicates the location of potential cleavage site along the Spike glycoprotein. Red dots represent the basic amino acids, potential protease cleavage sites (red letter) and two red arrows indicate the cleavage sites identified. NTD – N-terminal domain, RBD – receptor-binding domain, RBM – receptor-binding motif, FP – fusion peptide, HR-N – N-terminal of heptad-repeat, HR-C – C-terminal of heptad-repeat, IC – Intracellular tail.

The susceptibility of purified recombinant triSpike proteins to protease cleavage was investigated with airway proteases because SARS-CoV is pneumotropic. Protease cleavage on purified triSpike protein was performed by treating the triSpike protein with airway proteases followed by amino acid sequence analyses. Results indicated that different airway proteases recognize amino acid residues of Spike as cleavage sites (Figure 2A). We also observed additional bands, migrating between 75 and 150 kDa, which may represent either incomplete cleavage products or a contamination of the immunopurified triSpike. Amino acid sequences corresponding to the cleaved SARS spike were identified and sequenced (Figure 2A). The efficiency of protease cleavage activity was also studied by comparing the yields of cleavage products obtained from the HPLC analysis using synthetic peptides corresponding to the cleaved SARS spike sequence. Our results showed that trypsin recognize and cleave peptides at both position R667 and R797 more efficiently than the other proteases. TMPRSS11a cleave peptides at the position R667 more efficiently than R797 whereas plasmin cleaves peptides at the position R797 more efficiently than R667 (data not shown). Sequence analysis of cleavage products showed that trypsin, plasmin and TMPRSS11a proteases cleave the Spike glycoprotein at positions 667 and 797 (Figure 2B). Interestingly, amino acid residue 667 has been previously proposed to be the potential cleavage site in Spike generating the S1 and S2 subunits [32]. Because protease processing of coronaviruses surface glycoproteins into S1 and S2 subunits is important to activate the fusion between the viral envelope and the cellular membrane during virus entry step [18], [21], cleavage by trypsin, plasmin and TMPRSS11a proteases at amino acid residue 667 suggests the involvement of airway proteases during virus entry and fusion. Amino acid residue 797 is also sensitive to cleavage by trypsin, plasmin and TMPRSS11a proteases. This site, which lies within the S2 subunit region, could be important for its further rearrangement, possibly bringing the virus envelope and cellular membrane in close proximity to facilitate virus-cell fusion. Altogether, we have identified at least two amino acid residues which are sensitive to airway protease cleavage *in vitro* and may be important for *in vivo* virus entry and fusion.

### Effect of Airway Proteases on SARS-S Mediated Virus Entry

**Figure 3:**
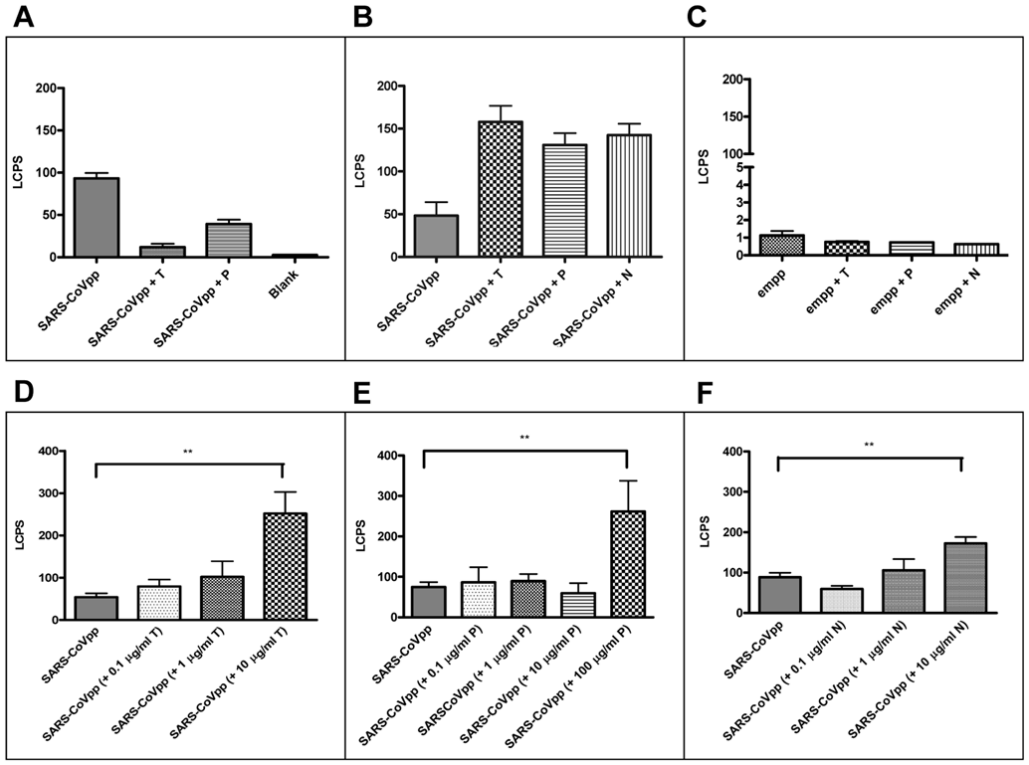
Effect of airway proteases treatment on SARS-CoVpp infectivity. A, SARS-CoVpp was pre-incubated with either trypsin (T) or plasmin (P) (10 µg/ml) at 37°C for 20 min. Luciferase activity (LCPS) was measured from infected 16HBE cells. Asterisk (*) indicates a value of *p*<0.05 in two-tailed *t* tests. Experiments were performed in triplicates and values were expressed as means±SE from two independent experiments. SARS-CoVpp entry into susceptible cell lines was enhanced with the presence of airway proteases. B & C, Equal amounts of SARS-CoVpp and empp (normalized to p24 quantity) were pre-incubated with 16HBE cells on ice for 30 min. Cells were washed twice to remove any unbound pp. Cells were incubated with 10 µg/ml of either trypsin (T), TMPRSS11a (N) or 100 µg/ml of plasmin (P) at room temperature for 40 min. Luciferase activity (LCPS) was measured from infected 16HBE cells. Experiments were performed in triplicates and values were expressed as means±SE from two independent experiments. D-F, Next, SARS-CoVpp was pre-incubated with 16HBE cells on ice for 30 min. Cells were washed twice to remove any unbound pp. Cells were incubated with various concentrations of trypsin (T), plasmin (P) or TMPRSS11a (N) at room temperature for 40 min. Luciferase activity (LCPS) was measured from infected 16HBE cells. Asterisk (**) indicates a value of *p*<0.01 in two-tailed *t* tests. Experiments were performed in triplicates and values were expressed as means±SE from three independent experiments.

We next investigated the effects of airway protease treatment on SARS-CoVpp entry into human airway epithelial cells (16HBE cells). Firstly, SARS-CoVpp was pre-treated with airway proteases and then incubated with 16HBE cells. We observed that proteolytic cleavage of Spike protein before virus attachment to cells had no enhancing effects on virus entry (Figure 3A). On the contrary, trypsin and plasmin treatment significantly reduced the infectivity of SARS-CoVpp (Figure 3A). According to this observation, it is most likely that protease treatment digested out the S1 region which is crucial for ACE2 binding, thus resulting in reduced infectivity of SARS-CoVpp.

Since attachment to virus receptor(s) may trigger critical conformational changes for proteolytic cleavage, we reasoned that it was necessary to investigate whether airway proteases would alter SARS-CoVpp entry after virus-cell attachment. 16HBE cells were pre-incubated with SARS-CoVpp (Figure 3B) or lentiviral vector without surface glycoprotein (referred to herein as empp) (Figure 3C) on ice, hence allowing virus attachment but not virus entry. Unbound SARS-CoVpp or empp was washed away and proteases were added. Under these conditions airway protease treatment significantly enhanced the infection by triSpike pseudotypes (Figure 3B). To verify if the concentration of airway proteases had any enhancement effects on virus entry, unbound SARS-CoVpp was washed away and various concentrations of proteases were added. The enhancement of SARS-CoVpp entry increased with higher concentration of the proteases (Figure 3D–F). Taken together, these observations clearly demonstrate that airway protease activity can modulate the efficiency of SARS-CoV entry into human ACE2-expressing airway epithelial cells, in a manner that is step-dependent on virus-cell attachment.

### Mutant SARS-CoV Spike Glycoprotein Affects Virion Incorporation

**Figure 4:**
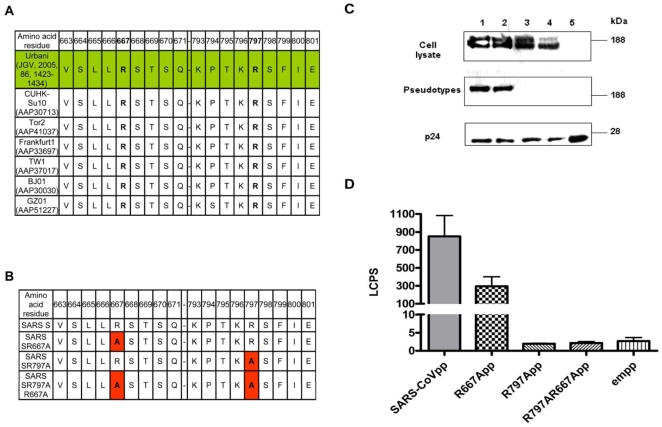
Differential expression of wild-type and mutant SARS S glycoprotein and SARS-CoV pseudotype production from 293T cells. A, Different putative spike glycoprotein sequences from SARS-CoV isolates were obtained from NCBI. Name of SARS-CoV isolates and GenBank accession numbers are listed. Potential airway protease cleavage residues are highlighted in green. B, Three mutant constructs were made from the wild-type SARS-CoV Spike glycoprotein cDNA cloned in the vector pcDNA3.1 as described in Materials and Methods. C, Cell lysates were collected from 293T cells 48 h post transfection. Pseudotypes were collected from culture medium and concentrated as described previously [33]. Samples (cell lysates or pseduotypes) were denatured, reduced and analyzed by 4–12% Bis-Tris SDS-PAGE gel and Western Blot using M2 monoclonal antibody against the FLAG peptide. Sizes of molecular weight markers are indicated on the right. Wild-type SARS spike (lane 1), R667A (lane 2), R797A (lane 3), R797AR667A (lane 4) and pseudotype without envelope (lane 5). D, Analysis of various types of SARS-CoVpp for viral entry. SARS-CoVpp (wild-type or mutant SARS S glycoprotein) were incubated with 16HBE cells and transduction was measured by determination of the luciferase activity (LCPS). Experiments were performed in duplicates and data are presented as means±SE from two independent experiments.

Multiple sequence alignments were done on different isolates of the SARS-CoV spike glycoprotein to locate the airway protease cleavage sites (Figure 4A). Clearly, the potential airway protease cleavage motif is relatively conserved between the various isolates with over 90% sequence identity. In order to study the functional role of the protease cleavage site, 2 alanine substitutions (either single or double mutation) were generated by site-directed mutagenesis (Figure 4B) and tested by pseudotyping lentiviral vectors with different mutants of SARS-CoV spike glycoprotein.

Expression of wild-type and mutant envelope proteins in transfected cells was tested by Western blot analysis (Figure 4C). Cell lysates expressing wild–type Spike showed the characteristic doublet of SARS spike glycoprotein (upper panel, lane 1), indicating that SARS spike glycoprotein is expressed and processed to incorporate high mannose or complex sugars [33]. In the case of the mutant plasmids, the expression levels and doublet patterns of SARS spike glycoprotein (R667A or R797A) in cell lysates suggest that envelope protein expression and post-translational modifications have not been significantly affected by any of the substitutions (Figure 4C; upper panel, lanes 2–3). By contrast, double mutation (R797AR667A) plasmids exhibited greatly reduced levels of complex-sugar linked envelope expression in the cell lysates, without apparent altering the addition of high-mannose sugars (Figure 4C; upper panel, lane 4). Possibly, the double mutation could have affected proper folding and oligomerization, which is important for the maturation of envelope glycoproteins.

We then aimed to establish whether mutant SARS spike glycoprotein can efficiently incorporate into SARS-CoVpp by Western blot analysis of pseudotyped particles purified (20% sucrose) from cells transfected with either wild-type or mutant plasmids. Proper virion incorporation requires surface expression of envelope glycoprotein, and is dependent upon correct folding and oligomerization [34], [35]. As shown in Figure 4C, assembly of co-transfected foreign proteins into *bona fide* pseudoparticles was tested by the presence of the HIV p24 protein of the lentiviral vector backbone in the same samples. The wild-type pseudotypes expressed the envelope glycoprotein as a single band (Figure 4C; middle panel, lane 1), suggesting that the envelope glycoprotein was incorporated into pseudotypes. The detection of complex-sugar (but not high-mannose) linked envelope in purified pseudotypes indicates that envelope glycoprotein incorporation requires the completion of all the quality-control processes along the secretory pathway [35]. In contrast, the double mutant (R797AR667A) exhibited a complete lack of pseudotype incorporation of envelope glycoprotein (Figure 4C; middle panel, lane 4), illustrated by the presence of p24 in the absence of mature envelope glycoprotein (Figure 4C; lower panel, lane 4). Surprisingly, the single mutants (R667A and R797A) exhibited differential patterns of envelope glycoprotein incorporation into pseudotypes (Figure 4C; middle panel, lanes 2–3). Both constructs displayed a band pattern similar to that of wild-type, suggesting that the envelope glycoprotein was expressed in transfected cells. However, only R667A was detected in purified pseudotypes. The absence of R797A envelope glycoprotein incorporation into the pseudotypes may be due to a disruption of folding and/or oligomerization along the secretory pathway.

The mutational effect on virus entry into 16HBE cells was then explored by measuring the activity of the luciferase reporter gene (Figure 4D). The R667A mutant was functional and exhibited virus entry activity similar to that induced by wild-type SARS-CoVpp, albeit at a slightly lower level (Figure 4D). As expected, R797A and R797AR667A mutants were unable to enter cells (Figure 4D, cf. with the empty vector). This observation correlates with the absence of envelope glycoprotein incorporation into the pseudotypes.

### Mutation of a Protease Cleavage Site Abrogates Virus Entry Enhancement

**Figure 5:**
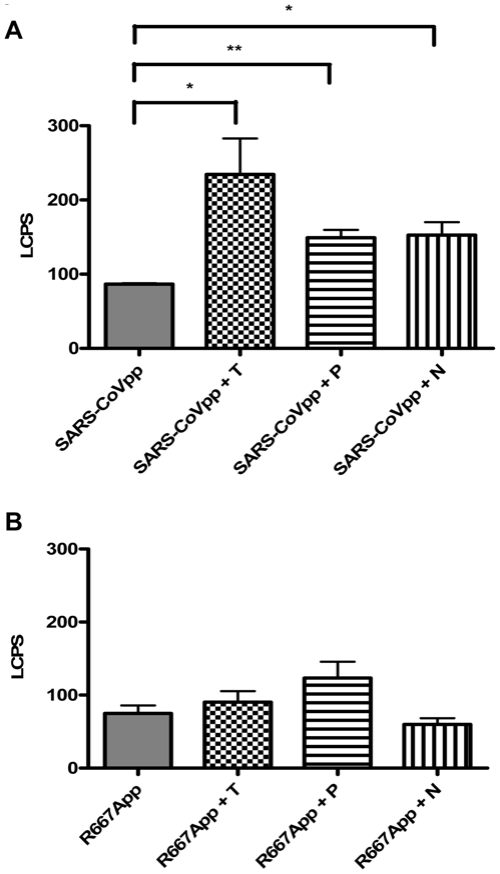
Role of amino acid residue 667 enhances SARS-CoVpp entry in the presence of airway proteases. SARS-CoVpp (A) or R667App (B) was pre-incubated with 16HBE cells on ice for 30 min. Cells were washed twice to remove any unbound pp. Cells were incubated with 10 µg/ml of either trypsin (T), TMPRSS11a (N) or 100 µg/ml of plasmin (P) at room temperature for 40 min. Luciferase activity (LCPS) was measured from infected 16HBE cells. Asterisk (*) indicates a value of *p*<0.05 and (**) indicates a value of *p*<0.01 in two-tailed *t* tests. Experiments were performed in triplicates and values were expressed as means±SE from three independent experiments.

In an effort to directly demonstrate that airway protease mediated virus entry enhancement is due to the presence of cleavage site on the SARS spike glycoprotein, 16HBE cells were pre-incubated with wild-type (SARS-CoVpp) or mutant (R667App) pseudotypes on ice, which allowed virus attachment but not entry. Unbound pseudotypes were washed away and proteases were added. Our results showed that both the wild-type and mutant spike glycoproteins were functional and mediated virus entry into 16HBE cells (Figure 5A and 5B). Whereas airway protease treatment significantly increased the entry of SARS-CoVpp into susceptible cells (Figure 5A), this enhancing effect was completely abrogated with R667App (Figure 5B). Our data indicate that the arginine residue at position 667 is sensitive to airway protease cleavage which, in turn, significantly increases SARS-CoV infectivity. Airway proteases could cleave the SARS spike glycoprotein following the virus-cell attachment stage, thereby enhancing SARS-CoV entry into human ACE2-expressing airway epithelial cells *in vitro*.

## Discussion

The spike glycoproteins of many enveloped viruses, including various strain of human influenza viruses and Sendai virus are cleaved by host-derived proteases into two non-covalently linked subunits [5], [36]. Proteolytic modification of spike glycoproteins is the major determinant of virus tropism and pathogenicity as shown in pneumotropic viruses whose infectivity is determined by airway proteases [5], [36], [37]. A monobasic cleavage site has been identified in various viral glycoproteins and is recognized by proteases secreted by epithelial cells [2]. Our data demonstrate that SARS spike glycoprotein has two monobasic cleavage sites that are susceptible to airway protease cleavage. We show here that specific airway proteases secreted along the respiratory tract, such as trypsin, plasmin and TMPRSS11a, recognize and cleave the same two monobasic motifs on the SARS spike glycoprotein. Furthermore, we have taken advantage of the flexibility of pseudotyped particles to investigate the molecular mechanisms underlying initial steps of virus entry following cell attachment. We have observed that protease cleavage of spike glycoprotein greatly enhances entry but only after receptor-binding interaction at the cell surface. These observations suggest that proteolytic processing of bound spike results in a conformational change that favors entry of the virus. This is the first report which demonstrates the functional impact of disrupting cleavage at position 667, which had not been addressed before. More importantly, it clarifies the mechanism of virus entry enhancement by several airway proteases. Our data are in agreement with the recent study showing that SARS-CoV S-mediated virus entry is dependent on the sequential proteolytic cleavage of monobasic sites [27].

It has been shown in clinical observations from SARS-infected patients' samples that the respiratory tract is the major cellular target for infection and replication [28], [29]. Similar to other coronaviruses, SARS-CoV utilizes the respiratory and gastrointestinal tract system as the primary entry site for infection. However, unexpected findings from several studies indicated that human airway epithelial cell lines were non-permissive to SARS-CoV [30], [38], [39]. We found that the permissiveness of SARS-CoV depended on ACE2 expression, further confirming earlier studies which identified ACE2 as the functional cellular receptor for SARS-CoV entry [40], [41]. SARS-CoV was also detected from lungs, gastrointestinal tract and kidney autopsy samples [42], correlating tissue tropism with the pattern of ACE2 expression in humans [43].

Interestingly, ACE2 expression on epithelial cell was found to correlate with the levels of cellular differentiation [31], and primary cells grown under air-liquid interface stimulated cellular differentiation and apical surface expression of ACE2 [31]. Although 16HBE and BEAS-2B cells are derived from human bronchial epithelium [44], [45], they showed unique morphological and biochemical characteristic according to their specific levels of cellular differentiation [46], [47]. Our study demonstrate another unique characteristic within these two cell lines in terms of cell surface protein expression, further establishing 16HBE cells, which have ACE2 expression under normal culturing system, as an *in vitro* model for SARS-CoV study.

Pseudotyped viruses have been useful for studying virus entry mechanism, cell tropism, neutralizing antibody, and receptor identification [48], [49], [50]. We have observed differential virion incorporation, which is dependent on surface expression of envelope glycoproteins, correlating with the proper folding and post-translational modification of the envelope glycoprotein [35]. Our data is consistent with previous studies indicating that SARS spike producing cells express both the high-mannose and complex-sugar linked envelope glycoprotein [33], and showed that complex-sugar linked SARS spike glycoprotein is solely incorporated into SARS-CoVpp. The SARS spike double mutant (R797AR667A) had defects in virion incorporation, likely due to the impaired post-translational modification of the envelope glycoprotein. Surprisingly, even mutant R797A showed a lack of virion incorporation. We postulate that the defect in this case is due to the impaired oligomerization of envelope glycoprotein. Amino acid residue 797 is located within the S2 region of SARS spike glycoprotein, which is important for trimeric envelope glycoprotein formation (unpublished observations). As a result, any mutations at this residue might generate localized folding defects of the envelope glycoprotein, and cause reduced levels of stable surface expression that prevent efficient incorporation into pseudotypes. A separate study has recently demonstrated the incorporation of a similar mutant construct (R797N) into a murine leukemia virus (MLV)-based pseudotyping vector [27]. The difference in mutant envelope incorporation with respect to our study might be attributed to the greater hydrophobicity of our substituted amino acid, which disrupts the oligomerization process. Clearly, further investigation will be required to assess the role of this amino acid residue in SARS spike oligomerization.

Previous studies have demonstrated the effects of intracellular proteases treatment (e.g. cathepsin L) in activating the membrane fusion property of SARS spike [51], [52]. For example, an important role of R797 cleavage site has been shown by artificially inserting a furin cleavage site, which resulted in the production of cleaved spike glycoprotein pseudotype, and allowed the infection of cells in the presence of protease inhibitors [52]. However, wild-type SARS spike has no putative furin cleavage motif/site. In addition, SARS-CoV has primarily respiratory tropism where a number of extracellular proteases are secreted by the airway epithelium. Therefore, our results suggest that, extracellular airway proteases localized along the respiratory tract cleave and modify the wild-type SARS spike during the very early stage of virus entry (viz., virus-cell attachment step). This modification enables the formation of a cleaved form of spike, and facilitates the activation of membrane fusion by subsequent intracellular protease treatment. It remains possible that the sequential activation of extracellular and intracellular proteases facilitates SARS-CoV entry along the respiratory tract. Another aspect which should be considered is that the cleavage sites described in this report were identified using soluble spike protein, and it is conceivable that additional sites may be involved upon receptor binding. However, the impact of the R667A mutation clearly indicates the functional importance of cleavage at this position for virus entry.

One study also showed an effect of very large concentrations of proteases in enhancing SARS-CoV infection on VeroE6 cells [24]. However, our data demonstrates that the effective concentrations of airway proteases inducing SARS-CoV entry enhancement was at least 100-fold less than that previously reported [24]. This difference could be due to the duration of airway protease treatment onto virus-adsorbed cells, the cleavage efficiency of airway proteases or the sensitivity of the detection system used. Of note, we found that the enhancing effect of airway proteases on SARS-CoVpp entry is strictly dependent on the virus-cell attachment step.

Our findings are in contrast with earlier studies done on coronavirus MHV-A59 where proteolytic cleavage of envelope glycoprotein on virion surface, before binding to its cellular receptor, is necessary for entry and fusion process [18], [21]. We clearly observed that pre-treatment of SARS-CoVpp with airway proteases dramatically reduced the infectivity of pseudotype particles, whereas the addition of airway proteases after virus-cell attachment enhanced virus-cell fusion. We postulate that receptor-bound spike glycoprotein is required for cleavage mediated virus entry enhancement, as the spike glycoprotein undergoes conformational changes once it binds to the cellular receptor. It is believed that envelope glycoprotein undergoes multiple programmed conformational changes during cellular receptor interaction [53], [54], [55]. This process will expose the hydrophobic fusion peptide and mediate fusion of the viral envelope with the host cell membranes [56]. We have shown, however, that entry of SARS-CoVpp could still complete without addition of airway proteases. Several possibilities could account for this finding. First, SARS-CoVpp could enter 16HBE cells through an endosomal pathway as described previously [23]; alternatively, there could be a partial switch of spike glycoprotein from inactive to active conformation upon receptor binding. We hypothesize that, in the presence of airway proteases, cleavage of receptor-bound spike glycoprotein might reduce the threshold for conformational change of spike glycoprotein into its fusogenic form, thus facilitating the exposure of fusion peptide and its interaction with host cell membranes.

Current studies have suggested that SARS-CoV enters and exits preferentially via the apical surface of the epithelium [31], and co-localization of airway proteases with SARS-CoV along the respiratory tract supports the positive feedback loop of virus infection *in vivo*. To conclude, we have found that cleavage of the receptor-bound spike glycoprotein by airway proteases enhances *in vitro* virus entry and fusion. Therefore, identification of reagents that are able to suppress *in vivo* activity of airway proteases might provide additional antiviral strategy against SARS-CoV infection [57], and possibly other viral respiratory infections such as human influenza A virus in the face of the current flu epidemic threat.

## Materials and Methods

### Cell Lines and Expression Vectors

HEK293T and VeroE6 cell lines (ATCC) were cultured at 37°C, 5% CO_2_, in Dulbecco's modified Eagle medium supplemented with 10% FBS, 100 U/ml penicillin, and 100 µg/ml streptomycin. Transformed human alveolar basal epithelial A549 (ATCC) and transformed bronchial epithelial BEAS-2B [50] cell lines were cultured at 37°C, 5% CO_2_, in F12K medium supplemented with 10% FBS, 1% L-Glutamine, and 1% antibiotic/antimycotic. Transformed bronchial epithelial 16HBE cells [49] were cultured at 37°C, 5% CO_2_, in Dulbecco's modified Eagle medium supplemented with 10% FBS, 1% L-Glutamine and 1% antibiotic/antimycotic. Complete medium was sterilized using 0.22 µm filtering units (Corning). SARS-CoV Spike cDNA and tagging with the C-terminal M2-FLAG peptide sequence (S-FLAG) have been described previously [58]. For improved expression yield, a codon-optimized SARS-S DNA containing a FLAG sequence fused in-frame at the 3′ end was produced using GeneOptimizer Technology (Geneart). Codon-optimized S-FLAG was subcloned into pcDNA3.1 expression vector resulting in the expression plasmid pcDNA-S-FLAG.

### Western Blot Analysis

Cell lysates were collected from 293T producing cells in lysis buffer (20 mM Tris-HCl pH 7.5, 150 mM NaCl, 2 mM EDTA, 1% Triton X-100). Virus pellets were prepared by ultracentrifugation on a 20% sucrose cushion at 140,000 x g for 3 h using a Beckman SW32Ti rotor and resuspended in TNE buffer (50 mM Tris-HCl, 100 mM NaCl, 0.5 mM EDTA; pH 7.4) before being subjected to SDS-PAGE (4–12%). Proteins were transferred onto nitrocellulose membranes, which were first incubated in blocking solution (PBS, 0.1% Tween 20, 5% milk powder, 3% Normal Goat serum), and then probed with anti-FLAG M2 monoclonal antibody (Sigma). HIV-1 p24 was detected by HIV-1 p24 antibody (abcam®). Peroxidase-conjugated goat anti-mouse IgG (H+L) (Zymed) was used as the secondary antibody. Bands were visualized on X-ray films (Kodak) using chemiluminescence (Amersham Biosciences).

### ACE2 Expression by Reverse Transcriptase PCR (RT-PCR)

Total mRNA was isolated from VeroE6, 16HBE, BEAS-2B and A549 cells using Qiagen RNEASY Midi total RNA extraction kit according to the manufacturer's instructions and dissolved in 60 µl of RNAse free H_2_O. ACE2 cDNA was synthesized using the ThermoScriptTM RT-PCR kit with ACE2 specific primers (Forward primer: 5′-gca ctc acg att gtt ggg act-3′, Reverse primer: 5′-att agc cac tcg cac atc ctc-3′) and amplified using REDTaq DNA Polymerase (Sigma). The quantity of total RNA templates was normalized to β-actin expression (Forward primer: 5′-gct cgt cgt tcg aca acg gct c-3′, Reverse primer: 5′-caa aca tga tct ggg tca tct tct c-3′).

### Cloning and Site-Directed Mutagenesis of SARS Spike Glycoprotein

Airway protease cleavage site mutants were prepared from plasmid pcDNA-S-FLAG with the Stratagene QuikChange site-directed mutagenesis kit. Site-specific mutagenesis were carried out by a single-step polymerase chain reaction using gene specific primers encompassing the site to be mutated and carrying the desirable mutated nucleotide(s). Methylated, non-mutated parental DNA template was removed by DpnI (10 U/µl) treatment and plasmids carrying the mutated nucleotide(s) were transformed into DH5α by electroporation. Positive clones carrying the mutated plasmids were screened by digesting the mutagenized pcDNA-S-FLAG sequences with unique restriction enzymes (MscI for R667A; StuI for R797A; MscI and StuI for R797AR667A). Mutations were verified by DNA sequencing.

### Cleavage and Peptide Mapping of S Protein by Airway Proteases

SARS spike glycoprotein (triSpike) was prepared as described previously [33]. The amount and degree of purity of recombinant triSpike protein was assessed by Western Blot and silver staining, respectively, as described previously [28]. Trypsin was purified from rat lung as described [11]. Plasmin was purchased from Calbiochem. Recombinant TMPRSS11a was kindly provided from Dr. Noboru Yamaji and Masako Kagoshima (Astellas Pharma Inc). Protease cleavage on purified triSpike protein was performed by treating the triSpike protein with airway proteases followed by amino acid sequence analyses. Purified triSpike protein was first incubated with various proteases including trypsin (1 µg/ml), TMPRSS11a (0.1 mg/ml) and plasmin (0.1 mg/ml) (equivalent to 0.2 mU of each proteases) in the presence of 0.1 M Tris-HCl, pH 7.5 at 37°C for 2 h. Purified triSpike protein incubated without proteases was included as control. Samples were then subjected to SDS-PAGE, performed on gradient acrylamide gels (2–15%) under reducing conditions according to the method of Laemmli [59]. Proteins were then transferred to PVDF membranes, which were stained with amino black or CBB to visualize cleavage products. After staining, protein bands were cored out and analyzed by N-terminal amino acid sequence determination using the Applied Biosystems 492 gas-phase sequencer/140C system, according to the manufacturer's instructions.

### Susceptibility of Human Airway Epithelial Cell Lines to SARS-CoV Infection

Protease cleavage assays were performed by treating SARS-CoVpp with airway proteases during the infection steps. Recombinant SARS-CoVpp lentiviral vectors expressing a luciferase reporter gene were produced from HEK293T cells as described previously [60] using 10 µg of plasmid pNL4.3.Luc.R^-^E^-^pro-viral genome [61], [62] and 10 µg of plasmid pcDNA-S-FLAG. For virus entry assays, VeroE6, 16HBE, BEAS-2B and A549 cells were infected with SARS-CoVpp. VeroE6 cells were seeded onto 96-wells plates at 10,000 cells/well in triplicates. 16HBE, BEAS-2B and A549 cells were seeded onto 96-wells plates at 20,000 cells/well, in triplicates and, the following day, 40 µl of SARS-CoVpp was added to each well. Cells were incubated for 1 h at 37°C in a 5% CO_2_ atmosphere, and then 160 µl of complete medium was added to each well for an overnight incubation. Medium was renewed 16 h later and incubation was continued for additional 48 h, after which cells were washed and lysed. Luciferase activity was measured by a MicroBeta Jet Counter (Perkin Elmer) according to the instructions provided by the supplier (Promega).

### Role of Airway Proteases on Viral Entry

To test the effect of airway protease treatment on virus entry, 16HBE cells were used as the model cell line for SARS-CoVpp infection. 16HBE cells were seeded onto 96-wells plates at 20,000 cells/well in triplicates. Before infection, cells were pretreated with reaction buffer (DMEM supplemented with 0.1 M Tris-HCl, pH 7.5) for 30 min on ice. SARS-CoVpp were added onto each well and incubated with the cells for another 30 min on ice. The mixture was removed and 100 µl of fresh reaction buffer was added to cells. Diluted airway proteases were added into each well and incubated for 40 min at room temperature. The mixture was removed; cells were washed twice with complete DMEM medium and incubated overnight at 37°C, in 5% CO_2_. Luciferase activity measurements were performed as described above.

### Statistical Analysis

Results are presented as means±SE of the specified number of samples from 2-3 independent experiments. Comparisons between two populations of data were made using the Student's unpaired *t*-test with a confidence limit for significance set at 0.05 or less.
